# Probing the Fractal Pattern of Heartbeats in Drosophila Pupae by Visible Optical Recording System

**DOI:** 10.1038/srep31950

**Published:** 2016-08-18

**Authors:** Chen Lin, Yi-Chung Chang, Ya-Chen Cheng, Po-Jung Lai, Chien-Hung Yeh, Wan-Hsin Hsieh, Kun Hu, June-Tai Wu, Hsiu-Hsiang Lee, Men-Tzung Lo, Yi-Lwun Ho

**Affiliations:** 1 Department of Biomedical Sciences and Engineering, National Central University, Taoyuan city, Taiwan; 2 Institue of Molecular Medicine, National Taiwan University College of Medicine, Taipei, Taiwan; 3 Division of Cardiology, Department of Internal Medicine, National Taiwan University Hospital and National Taiwan University College of Medicine, Taipei, Taiwan; 4 Medical Biodynamics Program, Division of Sleep and Circadian Disorders, Brigham and Women’s Hospital, Harvard Medical School, Boston, MA, USA; 5 Department of Electrical Engineering, National Central University, Taoyuan city, Taiwan

## Abstract

Judiciously tuning heart rates is critical for regular cardiovascular function. The fractal pattern of heartbeats — a multiscale regulation in instantaneous fluctuations — is well known for vertebrates. The most primitive heart system of the *Drosophila* provides a useful model to understand the evolutional origin of such a fractal pattern as well as the alterations of fractal pattern during diseased statuses. We developed a non-invasive visible optical heart rate recording system especially suitable for long-term recording by using principal component analysis (PCA) instead of fluorescence recording system to avoid the confounding effect from intense light irradiation. To deplete intracellular Ca^2+^ levels, the expression of sarco-endoplasmic reticulum Ca^2+^-ATPase (SERCA) was tissue-specifically knocked down. The SERCA group shows longer heart beat intervals (Mean ± SD: 1009.7 ± 151.6 ms) as compared to the control group (545.5 ± 45.4 ms, p < 0.001). The multiscale correlation of SERCA group (scaling exponent: 0.77 ± 0.07), on the other hand, is weaker than that of the control *Drosophila* (scaling exponent: 0.85 ± 0.03) (p = 0.016).

The mean number of heart beats throughout mammalian life span is proposed to be a constant. That is, the mammals with smaller size have shorter life expectancies due to their higher baseline heart rate corresponding to metabolism^1^. Human species, however, gains extra 10–20 years life expectancy with minimal increase of total lifetime heartbeats indicating that extension of human life does not achieved by simply slowing the heart rate. The evidence of a meta-analysis including around 68,000 patients also reveals that the patients with lower heart rate achieved by beta-blocker was associated with a higher risk for all-cause mortality, cardiovascular mortality or cardiovascular events^2^. From a holistic viewpoint, outputs from a wide variety of physiological systems, such as heart rate, exhibit multiscale regulation in complex temporal fluctuations^3^,^4^,^5^,^6^. In addition to numbers of heart beat, this regulation is characterized by fractal structures with properties that remain invariant over a wide range of time scales^7^. These fractal patterns are robust in healthy physiological systems but are significantly disrupted or abolished in degraded systems that are more vulnerable to catastrophic events and less adaptable to perturbations. Application of fractal analysis may provide new approaches to assessing cardiac risk and prognosis^8^,^9^,^10^,^11^,^12^,^13^. Therefore, it is believed that fractal is a hallmark of health physiological systems and that the underlying mechanisms of fractal regulation are of great interests but yet to be elucidated. We hypothesized that such fractal patterns of heart beats may be conserved in simple tubular structure of arthropods^14^. To explore this, we used heart tube of *Drosophila* as a model because it represents one of the most primitive heart systems in animal kingdom. Moreover, recent work indicates that release of Ca^2^+ into the cytosol from sarcoplasmic reticulum stores is critical to cardiac pacemaker in vertebrates and arthropod^15^,^16^,^17^ and clinically related to fatal arrhythmic events in patients with chronic heart failure^18^,^19^. The expression of sarco-endoplasmic reticulum Ca^2+^-ATPase (SERCA) was tissue-specifically knockdown to deplete intracellular Ca^2+^ levels, in this study.

To explore the time scale properties of the heart beat patterns for fractal analysis, especially for large time scale, hundreds of continuous heart beats are required in order to obtain reliable results. The usual way to detect heart beats in *Drosophila* is through the optical observing^20^. The structure of *Drosophila* heart can be examined using semi-intact preparations or a heart specific green fluorescent protein (GFP) marker (Cypher-GFP gene trap^21^). However, the semi-intact preparations disconnect heart tube from neighboring tissues thereby insulating heart tube from regulatory loops, which is the focus of our study. GFP marker, on the other hand, requires a high intensity light for visualization, which inevitably produces heat that may disturb cardiac function and heart beat patterns^22^,^23^. To detect *Drosophila* heart movement in an intact animal but free of thermal stresses, we take the images from the *Drosophila* under normal lamplight without dissection or exposure to laser radiations in the present study.

## Results

### Heart rate detection

**Figure 1:**
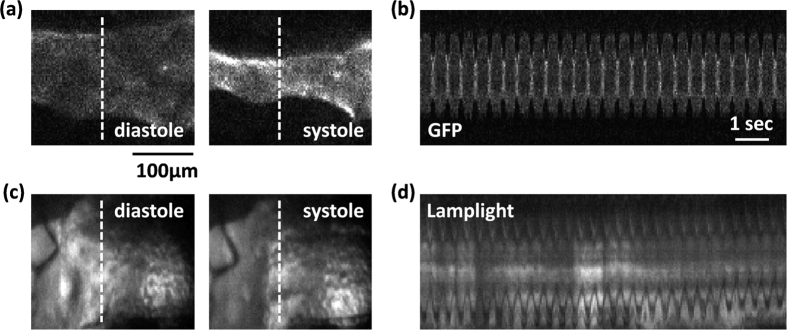
Recordings from systems using GFP marker and lamplights. (**a**) Diastole and systole image of *Drosophila pupae* heart with GFP marker. The dashed line represents one slice of heart tube. (**b**) The single pixel M-mode image represents the wall movements extracted from the dashed line. (**c**) Diastole and systole image of *Drosophila* heart under normal lamplight. The dashed line represents one slice of heart tube. (**d**) The single pixel M-mode image represents the wall movements extracted from the dashed line.

**Figure 2:**
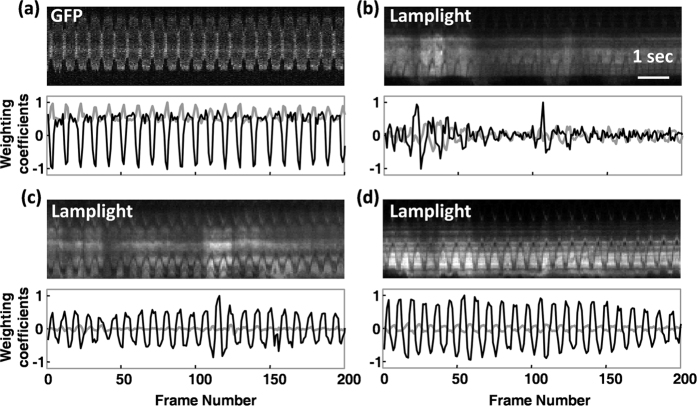
The wall motion of the heart extracted by PCA method. (**a**) The single pixel M-mode image of the heart with GFP marker and corresponding wall movements derived from PCA. (**b–d**) The single pixel M-mode image of the heart under normal lamplight and corresponding wall movements derived from PCA; the weighting coefficients suffer from poor image quality as indicated in (**b**).

Manifestation of *Drosophila* heart structure with GFP marker is shown in Fig. 1a. The wall motion of heart tube could be probed from the single pixel M-mode trace taken from the image sequence as shown in Fig. 1b. A typical *Drosophila* heart structure visualized under the normal lamplight is shown in Fig. 1c. The single pixel M-mode trace corresponding to different positions of heart tube are showed in Fig. 1d, for which the wall of heart is not readily identified as compared to that manifested with GFP marker. The extracted wall motion of the heart using principal component analysis (PCA) method is shown in Fig. 2. Although PCA is considered a powerful tool to reconstruct the temporal wall motion in the case with lamplight as indicated in Fig. 2b–d, the interference from other tissues can occasionally contaminate the extracted heart wall motion (Fig. 2b). To further minimize the effects of the contaminations to make the quality of reconstructed wall motion with lamplight be as good as that with GFP marker (Fig. 2a), we applied PCA to a number of wall motions that were reconstructed from different location of heart tube.

### Mean heart rate of SERCA and control animals

The mean cycle length of heart beats in the SERCA group was significantly longer as compared to the control group (control: 545.5 ± 45.4, SERCA: 1009.7 ± 151.6 ms, p < 0.001). The crawl activity is similar in SERCA and control *Drosophila larvae*, confirming a heart specific depletion of SERCA.

### Fractal correlations were presented in control group but were disrupted by the heart specific SERCA depletion

**Figure 3:**
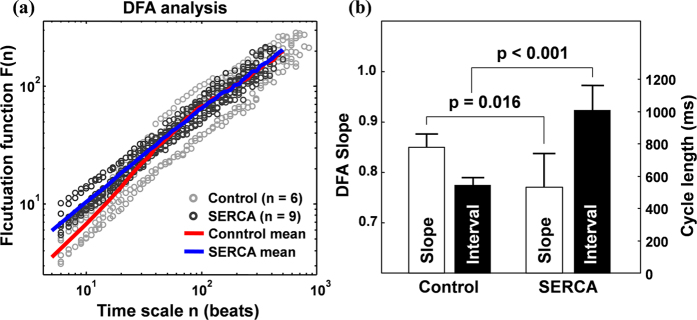
Analytical results of heart rate variation. (**a**) The fluctuation functions of control *Drosophila pupae* (gray circles and red line, n = 6, α = 0.85) and SERCA *Drosophila pupae* (black circles and blue line, n = 9, α = 0.77) are illustrated in a log-log plot; (**b**) Statistics of DFA slope and heart beat interval. The control group exhibited strong fractal correlations across a broad range of time scales from 6 up to 500 beats, as manifested by a straight line in a log–log plot, while those of the calcium depleted group were significantly reduced at time scales <500 beats.

To examine fractal patterns in heart beats fluctuations, we studied the correlation property in heart rate fluctuations from the control group by using the detrended fluctuation analysis (DFA). The heart rate fluctuations of the control group exhibited strong fractal correlations across a broad range of time scales from 6 up to 500 beats (~3–280 seconds), as indicated by a power-law form of F(n)~n^α^ (a straight line in a log–log plot) with α = 0.85 ± 0.03 (Fig. 3b). In contrast, fractal correlations were significantly reduced in the calcium depleted group at time scales <500 beats (α = 0.77 ± 0.07; p = 0.016) (Fig. 3b).

## Discussion

Studying the complexity of heart rate variability requires non-invasive instruments to conduct a long-term recording. Also, non-invasive modalities are preferable to preserve the intrinsic complexity of heart rate variability when we considered the possibility that the environmental factors, such as ambient temperature variations and thermal stresses, may disturb the control of cardiovascular function and metabolic regulation. While GFP marker has been widely adopted for the short recordings of the movement of heart contraction and the results are quite satisfactory, the accumulated heat generating by the high intensity light used for GFP excitation would induce either cardiomyopathy or irregular heart contraction^22^,^23^ and so limit its use for long-term recordings. To address this issue, we propose a label free method for the experiment. Using the new method, we extracted the rhythms associated with the heart contraction by applying PCA to the image series of resolution that is not sufficient for reliable detection of wall motion. The extracted rhythm faithfully recapitulated the cardiac cycles recorded from electrocardiogram and fluorescent image series. Importantly, it allows us to continuously monitor the heart rhythm over one hour, i.e., 12 times longer than the maximal duration allowed by conventional methods (~300 seconds for the electrocardiogram). Long-term and continuous recording of heartbeats will allow assessment of cardiac dynamics at large and multiple time scales that have been found to be of clinical relevance, being able to improve diagnosis of patients with heart diseases and to predict survival rate of these patients^24^,^25^,^26^.

One of the surprising findings in this study is that the regulation of the *Drosophila* heart shows a fractal character that persists for different gene types despite the difference in mean heart rate. The SERCA depletion certainly affects the rate of heart contraction but heartbeat fluctuations remain complex and possess fractal correlations over a wide range of time scales (6–500 beats). It is not clear whether fractal regulation of heartbeat fluctuations relies more on the intrinsic electrophysiological properties of a cardiomyocyte or structural properties of a beating heart as an entity. A fly’s heart tube is similar to the mammal counterpart at the cardiomyocyte level but extremely dissimilar in the structural level as an organ. Although the fly’s heart tube, composed of only 108 cardiomyocytes, is thousands fold less and simpler than the human heart, it still generates fractal heartbeat fluctuations similar to those observed in humans. This finding suggests that fractal regulation could be independent from the structural complexity of heart. This hypothesis is consistent with the fact that depleting SERCA in cardiomyocyte does not affect the anatomical integrity of heart tube but significantly affects fractal heartbeat fluctuations, indicating that intrinsic electrophysiological properties play a critical role fractal regulation of heartbeat fluctuations. It also provides one of the possible explanations that why the breakdown (decrease) of multiscale fractal correlation of the heart rate dynamics can be associated with increased risk of fatal arrhythmic events in different group of cardiovascular patients^8^,^9^,^24^ and yields prognostic values for the all-cause mortality of heart failure patients^26^.

We note that there was a large phase difference between optically and electronically measured rhythms in the previous study^27^. This phase difference may be resulted from a conductional delay from the origin of pacing to the imaged cardiomyocytes. The smooth waveforms on ultrasound Doppler and oxygen saturation meter still can be used for heart rate variability analysis^28^,^29^ on the large R-R variations. The extracted beat intervals show large variations even if the waveform is smooth. Furthermore, we simultaneously recorded the RR intervals derived from the electrode and our optical method. While the two methods showed consistent results of mean and standard deviation of RR intervals (Fig. S2), the optical method is suitable for the long-term recording without any complications. The results from DFA are neither random noise nor special pattern caused by artifacts, further supporting the validness of the variability calculated from these smooth waveforms.

Another concern is about the effects of heartbeat pause. It is well known that heart rate will dramatically increase when the ambient temperature goes up and vise versa. We observed that heart rate is reduced dramatically in SERCA group in our study, which is also found in other studies as well^17^. One possible explanation is that the *Drosophila* heart pause is the reaction to the mechanical stress^30^. In our study, we found that under GFP-labeled condition, the pause patterns will largely increase in both SERCA and control group that may be due to the exposure to the intense light. Moreover, the variation of heart rate in some SERCA larvae during simultaneous recording increased (Fig. S2b) especially in the end of recordings which suggests more irregular rhythmicity of the heart tube caused by the intrusive recording. Interestingly, neither the frequent occurrence of pauses nor the dramatic varying durations were witnessed in the long term spontaneous recordings without the external stimulations. Thus, the different pause patterns can be observed in each individual *Drosophila* irrelevant to the SERCA depletion which implies the spontaneous pause may not be affected by the SERCA depletion.

The cardiac morphology evolves from simple tubular structure in arthropods to multi-chambers in mammals, and the diversity is largely a matter of body size and adaptability^31^,^32^. However, whether the fractal heartbeat regulation is conserved or newly developed during evolution remains unknown, so does the molecular mechanism underlying heart rhythm variability as a physiological response to stimuli. Propagation of action potential, contraction of cardiomyocytes, and transcriptional program specifying heart development are similar between *Drosophila* and human, making *Drosophila* heart tube a feasible model to trace the origin of fractal cardiac dynamics. Complementarily, powerful fly genetics can allow us to explore the causal mechanisms of cardiac function at multiple time scales and from the molecular to system level.

## Method

### *Drosophila* strains, rearing and crosses

To deplete intracellular Ca^2+^ levels, we tissue-specifically knocked down the expression of SERCA^33^ by using GAL4-UAS bipartite expression system^34^. Specifically, a heart specific GAL4 line NP1029-GAL4^35^ is crossed with UAS-SERCA-dsRNA (Bloomington stock number 25928). The F1 progeny, NP1029-GAL4/+ (UAS-SERCA-dsRNA/+) expresses GAL4 transcription factor only in heart but not other muscle tissues to drive the expression SERCA-dsRNA, resulting in RNA interference (RNAi) mediated degradation of SERCA mRNA, reduced expression of SERCA, and depletion of sarco-endoplasmic reticulum calcium ion in cardiomyocytes. NP1029-GAL4/+ (UAS-mCD8-GFP/+) is used a control. Both sarco-endoplasmic reticulum calcium depletion SERCA and control flies were raised on standard sucrose-agar fly medium at 25 °C. For collecting images of heart tube under the normal lamplight, the transparent, immobile white *Drosophila pupae* within 30 minutes after its formation were subjective to image recording on standard glass slides without dissection and anesthesia.

### Experimental Setup

**Figure 4:**
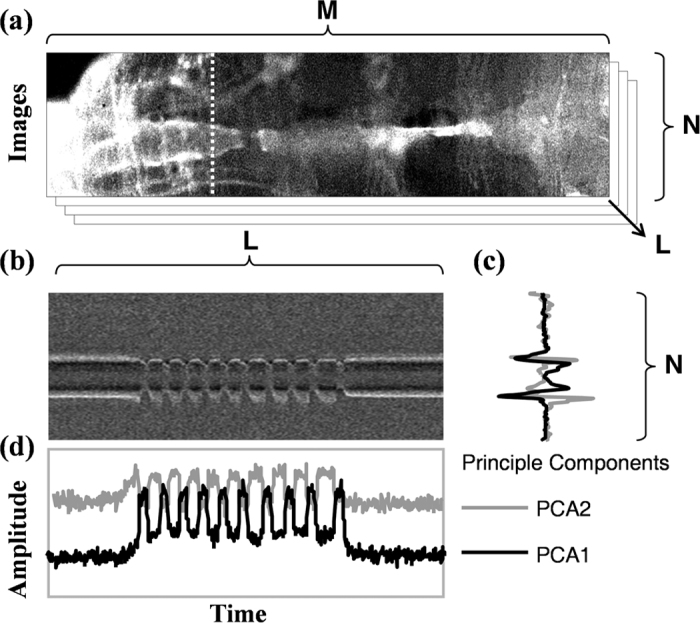
Ambiguous detection of heart tube wall with PCA method. (**a**) Original image of *Drosophila pupae* heart, dashed line represents one slice of heart tube, where M = 192, N = 128 and L = 100, and L is the time axis. (**b**) The single pixel M-mode image represents the wall movements extracted from the slice of heart tube (dashed line on Fig. 2a). (**c**) The two principal components obtained from PCA which are corresponding to heart contraction or relaxation. (**d**) Temporal variation of the contraction-relaxation ratios to the two fundamental principal components.

An individual larva was placed on a microscope slide, fixed by the double-sided tape and covered by a thin layer of glycerol to enhance transparency. Each sample was viewed through a Zeiss binocular microscope (SteREOLumar. V12, Carl Zeiss Microscopy GmbH, Germany) and images for heartbeat detection were continuously recorded under visual light for one hour (KL 1500 LCD, SCHOTT AG, Germany) (Fig. S1a). The long-term image was recorded in 6 normal and 9 SERCA Drosophila Pupae. Since the heart tube of Drosophila Pupae contracted segment by segment from posterior wall to anterior wall, we presumed that the contraction of the heart beat was initiated from the posterior pacemaker to pump out to aorta through cardiac valve of the heart tube. The cross-section of the image for beat detection was, therefore, selected from the region near the cardiac valve of the heart tube and the beat to beat heart rate intervals were derived by PCA method (see **Heartbeat sequence extraction**). The room temperature was maintained at around 25 °C and the body temperature of the larva (measured from the Infrared thermometer) was between 25 °C and 28 °C during the process. Streaming images of the beating heart were fed into a computer through a 100 Hz camera (5MP CMOS USB3.0, Mightex Systems, Canada) with resolution = 192 × 128 and exposure for 10 ms with 100 frame rate in a Grayscale color mode. Imaging processing and heartbeat detection software were coded by using Matlab software (Version 7.11, Mathwork Inc., USA). To verify the accuracy of the proposed method for heartbeat detection, the electrical signals of the heart tube on a different group of larva were simultaneously measured through the Tungsten electrodes. Note that the intrusive electrode observations will cause body fluid leakage and Drosophila death within few minutes. The electrodes were connected to a differential amplifier (ISO-80, World Precision Instruments, USA) and signals were digitized and recorded on a notebook computer as show in Fig. S1a. **Heartbeat sequence extraction.** PCA was utilized to detect heart tube wall from the video collected from camera. Video data were saved as a three dimensional *M* × *N* × *L* matrix, where *M* × *N* for horizontal and vertical direction of each image, and *L* for the number of image sequence (Fig. 4a). We applied PCA to the single pixel M-mode trace and derive from the movement matrix А (*N* × *L*) for a fixed horizontal position, which consists of selected slice (dashed line on the Fig. 4a) of image at different time to get two components associated with diastole or systole states. That is, A = *U*Σ*V*^T^, where the first and the second columns of the *U* matrix are the diastole or systole component, respectively (Fig. 4c). In addition, the first two columns of Σ*V*^T^ are the time function of weightings to the two fundamental principal components (Fig. 4d). The weighting coefficients of the first component can be associated with the heart wall motion. Since the motion of the heart tube was not always in the vertical direction, single pixel M-mode trace using a specific horizontal position might be interfered by occasional horizontal motion from other tissues. PCA was then applied to the matrix B which consists of temporal weighting function taken from different horizontal positions of heart tube to get dominant heart rhythmic contractions (major principal component of matrix B). With PCA, the wall motion of the heart derived from the continuous images under the normal lamplight was consistent with the electrocardiogram recording that was simultaneously measured through the Tungsten electrodes (Fig. S1b).

**Figure 5:**
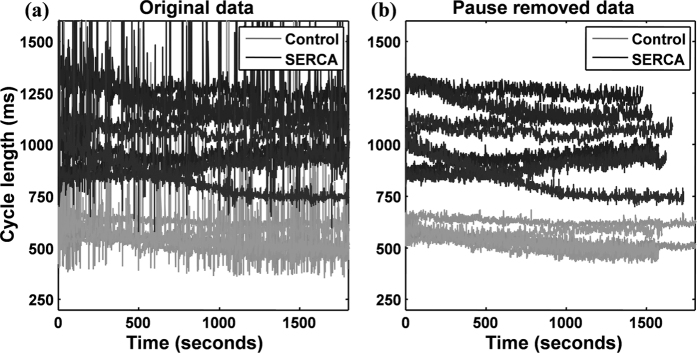
The difference between original and the de-noised heartbeat intervals in control and SERCA group. Beat to beat interval result, (**a**) the gray line represents the original heart duration sequence of control *Drosophila pupae* while the black one represents the original heart duration sequence of SERCA-RNAi *Drosophila pupae*; (**b**) The filtered results of both groups.

Finally, the local peaks of the reconstructed heart rhythmic contractions are identified and time series of peak interval can be taken as heartbeat fluctuations. Note that pauses (no contraction of the heart) often occurred in a long-term recording of a larva. Data during the paused periods were excluded for the further analysis (Fig. 5). To remove the outlier beats, a sliding window of the size of 10 beats was applied on the signal and an RR interval was identified as an outlier if it significantly deviated from the mean value of the window (i.e., 5 beats preceding and 5 beats following the RR interval). The degree of deviation used a predetermined threshold (e.g. 25% and 50% of the mean for upper and lower bounds, respectively)^36^. For each identified outlier, the value of that RR interval was linear interpolated from the two adjacent beats. The derived RR intervals were fairly consistent between heartbeats recorded by the proposed optical system and the electrogram method (Fig. S2).

### Detrended fluctuation analysis

To assess cardiac dynamics we performed DFA to estimate correlations in heartbeat fluctuations of a larva at different time scales^37^. Compared with traditional correlation analyses such as power spectral analysis and Hurst analysis, the DFA can accurately quantify correlations in data that may be masked by underlying nonstationarities or trends^38^,^39^. This method quantifies the detrended fluctuation function, F(n), of fluctuations at different time scales (heartbeat number n). A power-law form of F(n) indicates fractal regulation in the fluctuations, yielding F(n)~n^α^.The parameter α, called the scaling exponent, quantifies the correlation properties in the signal as follows: if α = 0.5, there is no correlation in the fluctuations (“white noise”); if α > 0.5, there are positive correlations, where large heartbeat intervals values are more likely to be followed by large activity intervals (and vice versa for small heartbeat intervals). Particularly, fluctuations with α ≈ 1 suggest very complex mechanisms that maintained fine balance between regularity and adaptability of systems, as observed in many physiological outputs under normal conditions.

### Statistical Analysis

The chi-square test or Fisher exact tests were used to compare nominal variables between groups. The continuous variables were represented as mean value ± SD and the normality of those variables was evaluated by using the Shapiro-Wilk test. The Mann-Whitney U test or Student’s t test was subsequently applied to the between-group comparison accordingly. All statistical tests were two-tailed and significance levels were set at p-values of less than 0.05.

### Limitations of the Study

One important limitation of optical measured rhythms must be borne in mind is that the derived RR intervals did not represent any information related to contractility or morphological changes of electrical waveforms. Although the depression of maximal force of contraction or alterations of myocyte action potential related to SERCA depletion is not main topic here, the longitudinal changes of those important parameters and their influences on the multiscale correlation related to SERCA gene warrant for further study. Finally, the algorithm for derive instantaneous RR interval from several seconds of data is needed for longer recording periods (e.g. hours to days) since the image data consumes too much storage space.

## Additional Information

**How to cite this article**: Lin, C. *et al.* Probing the Fractal Pattern of Heartbeats in Drosophila Pupae by Visible Optical Recording System. *Sci. Rep.* **6**, 31950; doi: 10.1038/srep31950 (2016).

## Supplementary Material

Supplementary Information
