# Supplementary material for: Probing the Fractal Pattern of Heartbeats in Drosophila Pupae by Visible Optical Recording System
Source: Sci Rep. 2016 Aug 18;6:31950. doi: 10.1038/srep31950 (PMC4989149; doi:10.1038/srep31950)
Supplement: Supplementary Information [file srep31950-s1.doc]

**Probing the Fractal Pattern of Heartbeats in Drosophila Pupae by Visible Optical Recording System**

Chen Lin, Yi-Chung Chang, Ya-Chen Cheng, Bor-Rong Lai, Chien-Hung Yeh, Wan‐Hsin Hsieh, Kun Hu, June-Tai Wu, Hsiu-Hsiang Lee, Men-Tzung Lo & Yi-Lwun Ho

**The setting of synchronous optical and electrocardiogram systems**

*For benchmarking the optical method, the electrodes were connected to a differential amplifier (ISO-80, World Precision Instruments, USA) and signals were digitized and recorded on a notebook computer. Both of the optical and electrical signal were synchronously sampled at 100 Hz. The contraction interval of the electrograms was detected by a threholding method and then the peak of each contraction was visually inspected to derive RR intervals of the electrogram.*

|  |
| --- |
| **Figure S1 Instrumentation with the Tungsten electrodes.** (**a**) Optical setup and synchronous electrocardiogram. (**b**) Heart beats obtained from PCA method and synchronous electrocardiogram. |

**Comparison of RR intervals between optical and electrogram systems**

The synchronous signals of optical and electrogram systems were recorded in 3 normal and 5 SERCA Drosophila pupae for 5 minutes. The voltage and waveform of electrical signals became weak and smoother at the end of most recordings. In addition to the delay between peaks (contraction of the heart tube) of electrical and optical signals (Fig S1b), the mean RR intervals were fairly consistent between the two methods as shown by Bland-Altman plot (Fig S2a). We further calculated the standard deviation (SD) of the RR intervals derived from the two methods. The SDs of the RR intervals in most of the Drosophila show good agreement between the two methods. However, the SD of RR intervals recorded from optical system in one SERCA was 13 ms larger than that from electrogram system causing by 5 to 6 beats with alternative shorten or prolong delays between electrogram and optical systems during recording (Fig S3). This discrepancy between electrogram and optical systems frequently accompany by decrease of the amplitude or morphologic changes of waveforms.”

|  |  |
| --- | --- |
| **Figure S2** Bland-Altman plot of difference in averaged RR intervals between the electrogram (RR_electrode_) and optical (RR_light_) systems (a) as well as the standard deviation of RR intervals between the two systems (b) for the 5-minute, simultaneous data**.** | |
|  | |
| **Figure S3** The synchronous signals of optical (upper) and electrograms (bottom) systems from the SERCA (-) Drosophila with (a) consistent delay of RR intervals and (b) alternative shorten or prolong delays between electrogram and optical systems during recording. The cross-section of the image used for beat detection was indicated by red arrow and the red line represents the synchronized frame time in electrogram system. | |
